# Postoperative chyle leak after pancreatic surgery: scoping review

**DOI:** 10.1093/bjsopen/zraf146

**Published:** 2026-02-09

**Authors:** Artur Rebelo, Enzo Rauchbach, Jörg Kleeff, Johannes Klose

**Affiliations:** Department of Visceral, Vascular and Endocrine Surgery, University Hospital Halle (Saale), Martin Luther University Halle-Wittenberg, Halle, Germany; Department of General, Visceral and Vascular Surgery, BG Hospital Bergmannstrost Halle, Halle, Germany; Department of Visceral, Vascular and Endocrine Surgery, University Hospital Halle (Saale), Martin Luther University Halle-Wittenberg, Halle, Germany; Department of General, Visceral and Vascular Surgery, BG Hospital Bergmannstrost Halle, Halle, Germany; Department of Visceral, Vascular and Endocrine Surgery, University Hospital Halle (Saale), Martin Luther University Halle-Wittenberg, Halle, Germany

**Keywords:** perioperative management, complications, therapy

## Abstract

**Background:**

Chyle leak is a significant complication after pancreatic resection, associated with increased morbidity and mortality. Data on its incidence, risk factors, and treatment are inconsistent. Robotic pancreatic resections are increasingly performed and assumed to be associated with fewer complications than open surgery. This study evaluated the incidence, risk factors, and therapeutic strategies for chyle leak after both open and robotic pancreatic surgery.

**Methods:**

A scoping literature review was conducted across multiple databases to identify studies that included patients who underwent open or robotic pancreatic resection and experienced chyle leak as defined by the International Study Group on Pancreatic Surgery. The search period extended from database inception until 27 August 2025.

**Results:**

In all, 58 studies published between 2007 and 2025 (30 039 patients) were included in the analysis. The pooled incidence of chyle leak after pancreatic resection was 8.0%. Procedure-specific pooled incidences of chyle leak were 9.5% after partial pancreatoduodenectomy, 8.4% after pylorus-preserving pancreatoduodenectomy, 6.9% after distal pancreatectomy, 1.7% after enucleation, and 6.2% after total pancreatectomy. In seven comparative studies (6339 patients), the pooled incidence of chyle leak was 10% after robotic pancreatoduodenectomy and 12% after open pancreatoduodenectomy.

**Conclusion:**

Chyle leak is an important complication following pancreatic resection. Despite advances in surgical techniques, the risk remains substantial, with no clinically significant difference in the rate of chyle leak between robotic and open pancreatoduodenectomy resections.

## Teaser

Chyle leak remains an important complication after pancreatic surgery, with a pooled incidence of 11.0%. This scoping review of 51 studies (23 565 patients) found no significant difference in the rate of chyle leak between robotic and open pancreatoduodenectomy. Risk factors include female sex, diabetes, and malignancy. Consistent treatment strategies are needed to mitigate associated morbidity.

## Introduction

According to the International Study Group on Pancreatic Surgery^1^, chyle leak is defined as the output of milky-coloured fluid from a drain, drain site, or wound on or after postoperative day 3, with a triglyceride content ≥ 110 mg/dl. Recent data suggest that chyle leak may occur in approximately one in eight patients after pancreatic resection^1^. A systematic review by Varghese *et al*.^2^ identified several risk factors for the occurrence of chyle leak after pancreatic resection, including patient-dependent and patient-independent variables. However, it should be noted that these data were obtained in the pre-robotic era and compared laparoscopic with open surgical approaches.

Robotic pancreatic resection is associated with several potential advantages, including lower rates of postoperative complications and therefore lower rates of postoperative morbidity^3^. Data from the EUROPA trail suggest no lower rates of chyle leak after robotic pancreatoduodenectomy^3^. However, the incidence of chyle leak after robotic pancreatic resection has not been investigated systematically as yet. Thus, the aim of the present study was to analyse the incidence of and risk factors for chyle leak after pancreatic resection, focusing on the comparison between open and robotic surgical approaches.

## Methods

### Search strategy

A literature search and data analysis were conducted according to the PRISMA extension for scoping reviews guidelines^4^ to identify studies that included patients who underwent open or robotic pancreatic resection and experienced lymphatic fistula. This study has been registered in the PROSPERO database (CRD42024575905).

A comprehensive literature search was conducted across the PubMed/MEDLINE, Web of Science Core Collection, Cochrane Library, and ClinicalTrials.gov databases (*Supplementary Figure 1*). Searches were performed via the respective online search engines and covered the period from database inception to 27 August 2025. The full search strategy is provided in the *supplementary methods*.

Following the literature search, all records identified were imported into reference management software, and duplicates were removed. Two reviewers (A.R., E.R.) independently screened titles and abstracts to identify studies relevant to the review question using predefined eligibility criteria. Records were categorized as ‘include’, ‘exclude’, or ‘unclear’. The full text of all potentially relevant or unclear records was retrieved and the articles were screened independently by the same two reviewers. Disagreements at any stage were resolved through discussion, and a third reviewer (J.Klo.) was consulted if consensus could not be reached.

The reference lists of all included studies were also manually screened to identify further potentially relevant publications. Targeted hand-searching and author-based searches were also conducted to capture additional studies that may not have been retrieved through the initial database search.

### Inclusion and exclusion criteria

Only articles published in English were considered for inclusion. Studies with patients undergoing pancreatic surgery (partial pancreatoduodenectomies, distal pancreatectomies, total pancreatectomies, and enucleations) that reported on the occurrence of lymphatic fistula were included. Review articles, case reports, case series with fewer than five patients, commentaries, and letters were excluded. In addition, patients who only underwent exploration were excluded from the analysis.

### Data collection

Data from the included studies were extracted separately by two authors (A.R., E.R.) and stored in a dedicated database. The following descriptive data were documented for each included study: the total numbers and subsets of pancreatoduodenectomies (standard and robotic), pylorus-preserving pancreatoduodenectomies, distal pancreatectomies, mixed procedures (if no specific type of pancreatic resection was documented), total pancreatectomies, and enucleations; demographic data, including patient sex and age; clinical variables such as diabetes status, length of hospital stay, operation time, and the occurrence of pancreatic fistulas; and oncological diagnosis regarding pancreatic head adenocarcinoma, cholangiocarcinoma, ampullary carcinoma, and duodenal adenocarcinoma, classified as total, positive, or negative for chyle leak.

In every study, the risk of bias was evaluated using the Newcastle–Ottawa Scale^5^.

### Statistical analysis

In line with the descriptive aims of this scoping review, pooled incidence rates for selected outcomes were calculated using a random effects model in Jamovi (version 2.3). Additional data handling and descriptive analysis were conducted using SPSS^®^ version 29 (IBM, Armonk, NY, USA). No quantitative synthesis was performed due to the high heterogeneity of the included studies.

## Results

**Fig. 1:**
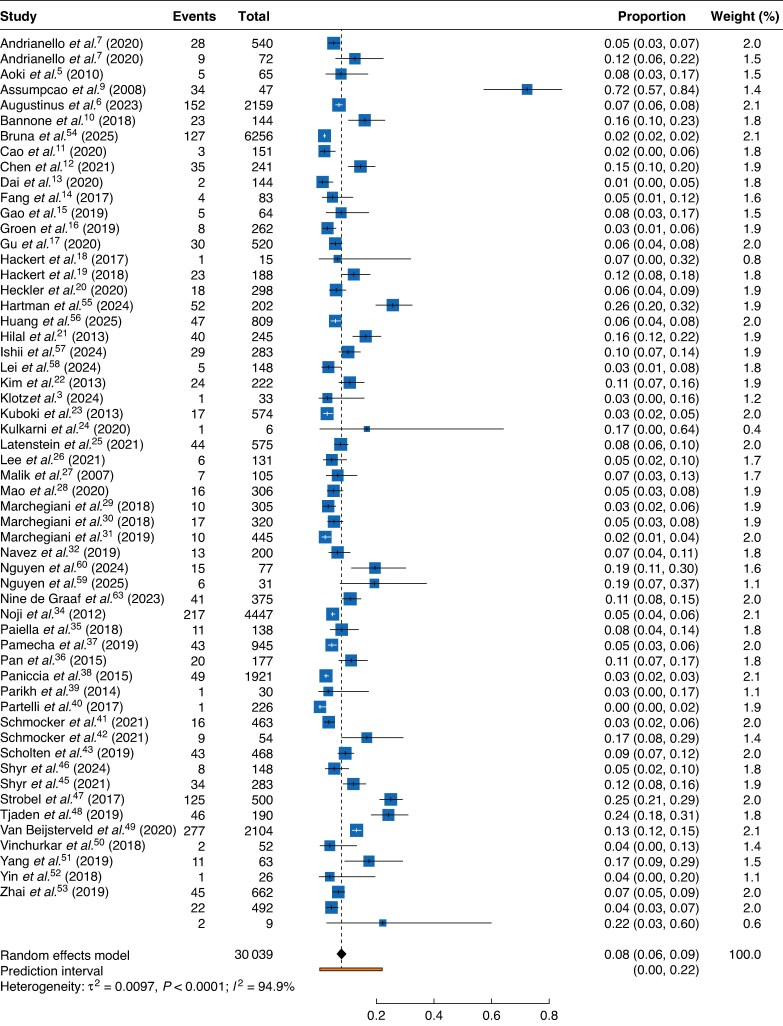
Pooled incidence of chyle leak after all pancreatic resections Values in parentheses are 95% confidence intervals.

After excluding non-relevant articles, 58 studies^3,6–62^ including 30 039 patients and published between 2007 and 2025 were reviewed and included in this analysis (*Table 1*). The reported incidence of chyle leak after pancreatic resection varied considerably across studies, ranging from 1 to 72%. The pooled incidence of chyle leak across all 30 039 pancreatic resections was 8.0% (*Fig. 1*).

**Table 1:** Studies included in the analysis

Study	Year	Inclusion period	No. of patients	Sex (*n*)		Mean age (years)	Diabetes (*n*)	Mean LOS (days)
				Male	Female			
Andrianello *et al*.^8^	2020	2017–2019	72	46	26	64	11	34
Andrianello *et al*.^9^	2020	2016–2019	540	304	236	63	76	
Aoki *et al*.^6^	2010	2001–2004	65					
Assumpcao *et al*.^10^	2008	1993–2008	3532			63.5		
Augustinus *et al*.^7^	2023	2017–2019	2159	1164	996	67.1		12
Bannone *et al*.^11^	2018	2016–2017	292	166	126	64	51	
Bruna *et al*.^55^	2025	2007–2020	6256					
Cao *et al*.^12^	2020	2010–2017	151	92	59	59	25	19.7
Chen *et al*.^13^	2019	2014–2018	241	143	98	64		15.7
Dai *et al*.^14^	2020	2014–2018	144	79	65	56.1	30	13.16
Fang *et al*.^15^	2017	2009–2014	83	55	28	70	14	19.35
Gao net al.^16^	2019	2009–2016	64	34	30	64		12.5
Groen *et al*.^17^	2018	2013–2017	262	133	129	63		9.5
Gu *et al*.^18^	2020	2014–2019	520	214	306	63		10.75
Hackert *et al*.^19^	2017	2016	15	7	8	60		
Hackert *et al*.^20^	2018	2013–2016	188	104	84	63.3	37	14.75
Hartman *et al*.^56^	2024	2020–2024	202					
Huang *et al*.^57^	2025	2016–2023	809					
Heckler *et al*.^21^	2020	2006–2014	298	128	170	66		
Hilal *et al*.^22^	2013	2007–2010	245	129	116	64.9	48	
Ishii *et al*.^58^	2024	2016–2022	283	141	142	76		
Lei *et al*.^59^	2024	2017–2022	148	92	58			
Kim *et al*.^23^	2013	2002–2010	222	115	107	61.3		25.05
Klotz *et al*.^3^	2024	2020–2022	62	33	29	63.6		15
Kuboki *et al*.^24^	2013	2000–2011	574	334	240	63.5		
Kulkarni *et al*.^25^	2020	2012–2019	6	3	3	43.6		
Latenstein *et al*.^26^	2021	2017–2018	575	315	260	68		
Lee *et al*.^27^	2021	2014–2020	131	69	63	66.5	46	
Malik *et al*.^28^	2007	1999–2005	105	59	61,5	61.5		
Mao *et al*.^29^	2020	2012–2014	306	186	120	62		
Marchegiani *et al*.^30^	2018	2014–2016	445	238	207	65		
Marchegiani *et al*.^31^	2018	2016–2017	320	167	153	62.9	53	11.4
Marchegiani *et al*.^32^	2018	2015–2016	91	51	40	63		16
Navez *et al*.^33^	2019	2008–2017	200	105	95	65		18
de Graaf *et al*.^34^	2023	2014–2021	1378	744	634	69		
Nguyen *et al*.^60^	2024	2020–2022	77	43	34	58.2	20	
Nguyen *et al*.^61^	2025	2021–2023	31	16	15	58.7		
Nickel *et al*.^62^	2024	2017–2022	375	212	163	64.9		
Noji *et al*.^35^	2012	1995–2010	138	60	78	66		
Paiella *et al*.^36^	2018	2014–2016	945	493	463	63	185	9
Pamecha *et al*.^38^	2019	2009–2018	177	131	46	57.1		
Pan *et al*.^37^	2015	2007–2013	1921	1218	703	58.5		
Paniccia *et al*.^39^	2015	2013–2014	30			63.1		
Parikh *et al*.^40^	2014	2007–2011	246	124	122	63	64	
Partelli *et al*.^41^	2017	2013–2015	463	261	202	68		
Schmocker *et al*.^42^	2020	2008–2018	54	25	29	62.7	4	
Schmocker *et al*.^43^	2020	2011–2018	468	222	246	65	125	
Scholten *et al*.^44^	2019	2006–2016	148			82	60	
Shyr *et al*.^45^	2020	2012–2017	283	129	129			24
Shyr *et al*.^46^	2021	2014–2019	451	240	211	65		
Shyr *et al*.^47^	2023	2012–2021	132	41	47	67		
Strobel *et al*.^48^	2017	2008–2012	2881				738	
Tjaden *et al*.^49^	2019	2001–2018	52	8	44	29	3	10
Van Beijsterveld *et al*.^50^	2020	2016–2018	63	31	32	68	6	14
Vinchurkar *et al*.^51^	2018	2012–2016	26	14	12	55		
Yang *et al*.^52^	2015	2009–2016	1921	1218	703	58.5		
Yang *et al*.^37^	2019	2006–2018	59	35	24	62		
Yin *et al*.^53^	2018	2012–2014	492	315	177	60.5		13.5
Zhai *et al*.^54^	2019	2017–2018	9	7	2	63.3	4	29

LOS, length of hospital stay.

### Demographic risk factors

Eight studies reported the sex-stratified incidence of chyle leak (*Table 2*). The incidence of chyle leak ranged from 3.4 to 15.5% in male patients and from 5.0 to 17.2% in female patients. Across studies, the direction and magnitude of differences between sexes were inconsistent: some studies reported slightly higher rates for female patients and others reported higher rates for male patients. No systematic sex-related trend could be identified. Given the heterogeneity in study design, surgical procedures, and outcome reporting, no pooled analysis was performed.

**Table 2:** Studies included in the analysis, with numbers of patients stratified according to demographic risk factors and diagnosis

Study	Male*	Female*	Pancreatic adenocarcinoma*	Cholangiocarcinoma*	Duodenal adenocarcinoma*	Ampullary carcinoma*	Other diagnosis*
Andrianello *et al*.^8^	46 (–)	26 (–)	21 (–)	9 (–)	9 (–)	11 (–)	20 (–)
Andrianello *et al*.^9^	304 (–)	236 (–)					
Aoki *et al*.^6^	− (4)	− (1)					
Assumpcao *et al*.^10^	− (25)	− (22)	− (32)	− (2)	− (4)	− (8)	− (1)
Augustinus *et al*.^7^	1164 (83)	995 (69)	1193 (83)	315 (23)	259 (21)	339 (20)	
Bannone *et al*.^11^	166 (–)	126 (–)	186 (–)	0	0	0	106 (–)
Bruna *et al*.^55^							127 (6256)
Cao *et al*.^12^	92 (–)	59 (–)					
Chen *et al*.^13^	143 (–)	98 (–)	109 (–)	65 (–)	11 (–)	31 (–)	25 (–)
Dai *et al*.^14^	79 (–)	65 (–)	72 (–)	6 (–)	6 (–)	9 (–)	32 (–)
Fang *et al*.^15^	55 (–)	28 (–)					
Gao net al.^16^	34 (–)	30 (–)					
Groen *et al*.^17^	133 (–)	129 (–)					
Gu *et al*.^18^	214 (–)	306 (–)	263 (–)	0	0	51 (–)	206 (–)
Hackert *et al*.^19^	7 (–)	8 (–)					
Hackert *et al*.^20^	104 (–)	84 (–)	80 (–)	0	0	0	108 (–)
Hartman *et al*.^56^	128 (–)	170 (–)	224 (–)	31 (–)	0	26 (–)	17 (–)
Huang *et al*.^57^							52 (202)
Heckler *et al*.^21^							47 (809)
Hilal *et al*.^22^	129 (20)	116 (20)	110 (19)	26 (6)	8 (2)	40 (3)	34 (6)
Ishii *et al*.^58^							29 (283)
Lei *et al*.^59^			5 (148)				
Kim *et al*.^23^	115 (9)	107 (15)	85 (8)	69 (7)	11 (1)	54 (7)	3 (1)
Klotz *et al*.^3^	33 (–)	29 (–)	22 (–)	3 (–)	0	3 (–)	5 (–)
Kuboki *et al*.^24^	334 (9)	240 (8)					
Kulkarni *et al*.^25^	3 (–)	3 (–)	2 (–)		0	0	4 (–)
Latenstein *et al*.^26^	315 (–)	260 (–)	310 (–)	152 (–)	14 (–)	99 (–)	0
Lee *et al*.^27^	69 (–)	63 (–)	19 (–)	45 (–)	3 (–)	29 (–)	36 (–)
Malik *et al*.^28^	59 (2)	61 (5)					
Mao *et al*.^29^	186 (–)	120 (–)	206 (–)	0	0	0	0
Marchegiani *et al*.^30^	238 (–)	207 (–)	445 (–)	0	0	0	0
Marchegiani *et al*.^31^	167 (–)	153 (–)	168 (–)	0	0	0	152 (–)
Marchegiani *et al*.^32^	51 (–)	40 (–)	42 (–)	2 (–)	1 (–)	7 (–)	39 (–)
Navez *et al*.^33^	105 (–)	95 (–)					
de Graaf *et al*.^34^	744 (–)	634 (–)	464 (–)	194 (–)	103 (–)	225 (–)	392 (–)
Nguyen *et al*.^60^							15 (77)
Nguyen *et al*.^61^							6 (31)
Nickel *et al*.^62^							41 (375)
Noji *et al*.^35^	60 (4)	78 (7)	59 (–)	0	0	0	79 (–)
Paiella *et al*.^36^	493 (20)	463 (23)	445 (18)	38 (1)	0	78 (3)	395 (21)
Pamecha *et al*.^38^	131 (–)	46 (–)	0 (–)	42 (–)	19 (–)	83 (–)	0
Pan *et al*.^37^	1218 (32)	703 (17)					
Paniccia *et al*.^39^							
Parikh *et al*.^40^	124 (–)	122 (–)					
Partelli *et al*.^41^	261 (–)	202 (–)					
Schmocker *et al*.^42^	25 (–)	29 (–)					
Schmocker *et al*.^43^	222 (–)	246 (–)					
Scholten *et al*.^44^			72 (–)	4 (–)	1 (–)	3 (–)	68 (–)
Shyr *et al*.^45^	129 (20)	129 (14)	96 (18)	0	0	0	187 (16)
Shyr *et al*.^46^	240 (–)	211 (–)	176 (–)	30 (–)	23 (–)	103 (–)	119
Shyr *et al*.^47^	41 (–)	47 (–)					
Strobel *et al*.^48^							
Tjaden *et al*.^49^	8 (–)	44 (–)					
Van Beijsterveld *et al*.^50^	31 (–)	32 (–)	42 (–)	0	0	0	21 (–)
Vinchurkar *et al*.^51^	14 (–)	12 (–)	0	0	0	26 (–)	0
Yang *et al*.^52^	1218 (–)	703 (–)	878 (–)	288 (–)	0	137 (–)	272 (–)
Yang *et al*.^37^	35 (–)	24 (–)	34 (–)	0	0	0	25 (–)
Yin *et al*.^53^	315 (–)	177 (–)	272 (–)	51 (–)	91 (–)	68 (–)	10 (–)
Zhai *et al*.^54^	7 (–)	2 (–)					

*The first value in each cell is the total number of patients, whereas values in parentheses indicate the number of patients with chyle leak. –, missing values.

### Diagnosis

Only a subset of studies reported pooled incidence stratified by diagnosis (*Table 2*). A subset of studies reported chyle leak incidence stratified by underlying diagnosis (*Table 2*). Among patients with pancreatic ductal adenocarcinoma, the reported incidence of chyle leak ranged from 3.5 to 18.8%. For cholangiocarcinoma, the incidence of chyle leak ranged from 4.3 to 15.1%, whereas rates of chyle leak in duodenal adenocarcinoma ranged from 5.3 and 18.4%. In patients with ampullary carcinoma, the incidence of chyle leak incidence ranged from 3.6 to 13.3%. Across studies, no consistent trend indicating higher or lower chyle leak rates by tumour type could be identified.

### Type of surgery

**Fig. 2:**
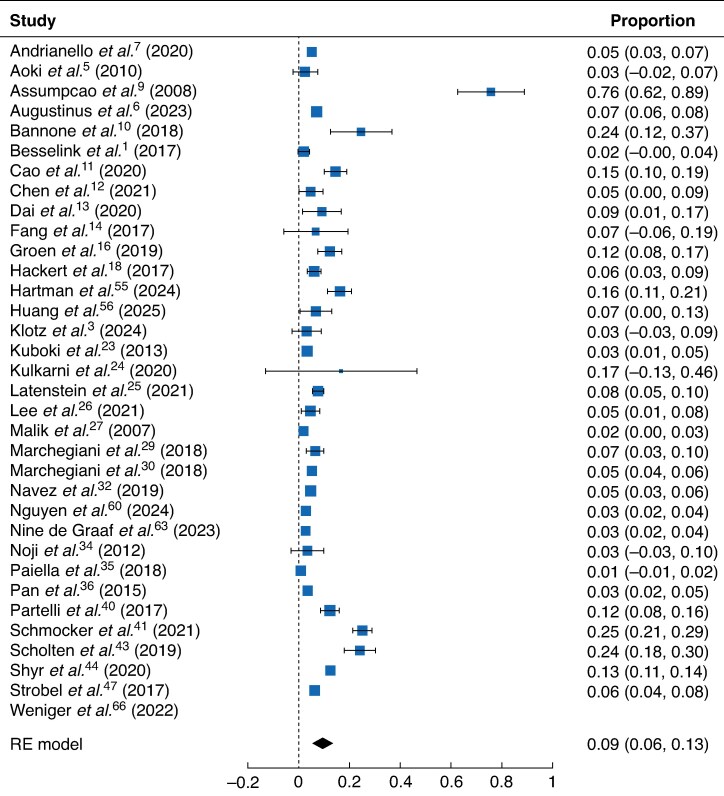
Pooled incidence of chyle leak after pancreatoduodenectomy Values in parentheses are 95% confidence intervals. RE, random effects.

The pooled incidence of chyle leak after partial pancreatoduodenectomy, as estimated using a random effects model, was 9.50% (*Fig. 2*). Patients from 33 studies were included in the analysis. The incidence of chyle leak after pylorus-preserving pancreatoduodenectomy was reported in a smaller subset of studies (*Fig. S2*); among these patients, the pooled incidence of chyle leak was 8.36%. The pooled incidence of chyle leak, estimated using a random effects model, was 6.89% (*Fig. S3*) after distal pancreatectomy, 1.70% after enucleation (*Fig. S4*), and 6.22% after total pancreatectomy (*Fig. S5*).

### Robotic pancreatoduodenectomy

Seven studies published between 2020 and 2025, including a total of 6339 patients, compared chyle leak after robotic *versus* open pancreatoduodenectomy. The pooled incidence of chyle leak across these studies was 11%.

**Fig. 3:**
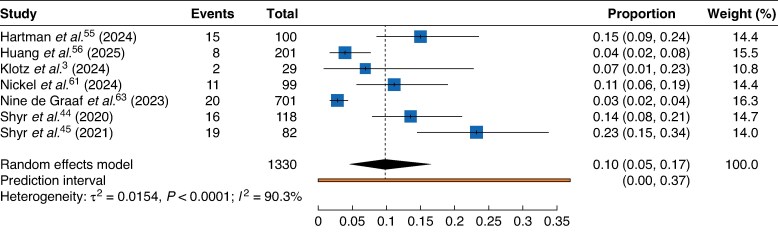
Pooled incidence of chyle leak after robotic pancreatoduodenectomy Values in parentheses are 95% confidence intervals.

Across seven studies including 1330 patients who underwent robotic pancreatoduodenectomy, the reported incidence of chyle leak ranged from 3 to 23%. The pooled incidence of chyle leak in this group was 10% (*Fig. 3*), whereas among the 5739 patients in the open surgery group, the pooled incidence was 12%.

## Discussion

The aim of the present comprehensive scoping review was to investigate the incidence of chyle leak after open and robotic pancreatic surgery. This study revealed that chyle leak is a frequent complication after pancreatic surgery, independent of the type of resection, and that robotic pancreatic resection is not associated with a clinically significant reduction in the rate of postoperative chyle leak.

There is no doubt as to the importance of chyle leak as a major complication after pancreatic surgery. Chyle leak may result in extended hospitalization periods, leading to increased healthcare costs and the consumption of medical resources. In addition, the initiation of adjuvant therapies may be delayed in patients with chyle leak after pancreatic surgery, resulting in impaired long-term outcomes^63^.

Further analysis revealed that the pooled incidence of chyle leak was higher after partial pancreatoduodenectomy, followed by distal pancreatectomy, total pancreatectomy, and finally enucleation. Strikingly, no significant interprocedural differences in the incidence of chyle leak were observed. In 2017, Strobel *et al*.^48^ reported that among more than 3000 patients, distal pancreatectomy was associated with a higher risk of postoperative chyle leak than other resections (*P* = 0.001). These data were quite surprising given that it was assumed that partial pancreatoduodenectomy would be associated with the highest risk of postoperative chyle leak^10^.

Minimally invasive pancreatic surgery, especially robotic-assisted surgery, raised hopes of a reduction in complications associated with the open approach. In the EUROPA trial^3^, a single-centre randomized clinical study, equivalent rates of chyle leak were reported after robotic partial pancreatoduodenectomy and open surgery, similar to the findings of a Dutch retrospective observational cohort study^7^. In another Dutch study, which compared only robotic and open partial pancreatoduodenectomy, revealed a trend towards lower rates of postoperative chyle leak after robotic resection^34^. Shyr *et al*.^64^ reported no differences in the incidence of chyle leak after robotic *versus* open partial pancreatoduodenectomy. Given that the surgical technique may not influence the risk of postoperative chyle leak, it is possible that the underlying disease might. This could also be explained by the fact that robotic partial pancreatoduodenectomy is reserved for relatively smaller tumours that do not require vascular resection. Only limited data on the extent of lymphatic tissue clearance during robotic pancreatoduodenectomy have been reported. The triangle operation is defined as complete removal of any lymphatic tissue between the portal vein, the coeliac trunk, and the superior mesenteric artery^45^. Data on the incidence of chyle leak are inconsistent, but extended resections appear to be associated with a higher risk of chyle leak^65^. Another recent study demonstrated that minimally invasive surgery is a risk factor for postoperative chyle leak after pancreatic resections^66^. Strobel *et al*.^48^ identified pancreatic ductal adenocarcinoma as a risk factor for postoperative chyle leak (adjusted hazard ratio 2.03; *P* < 0.001). Malik *et al*.^28^ found no significant effect of T or N tumour stage on lymphatic fistula formation, whereas Shyr *et al*.^45^ reported a significantly (*P* = 0.019) higher rate of lymphatic fistulas in patients with pancreatic head adenocarcinoma. Lymph node metastases, the number of lymph nodes removed, and the number of positive lymph nodes were identified as individual risk factors for lymphatic fistula formation.

Several studies reported that vascular resection during surgery increased the risk of lymphatic fistulas. The duration of surgery also emerged as a significant factor, with longer operations linked to higher risks^10^.

Therapeutic strategies for chyle leak were not analysed in this study. Conservative treatment and a fat-reduced or fat-free diet are therapeutic options for patients with grade A or B chyle leak^28^. Therapeutic lymphography may be an option for patients with grade C chyle leak, with encouraging data from a single-centre experience^67^.

This scoping review has several limitations. This analysis included not only on randomized clinical trials but also observational studies, causing heterogeneity in outcome definitions and treatments. The retrospective design across studies enhanced the risk for potential selection bias. The results stem from non-randomized, uncontrolled comparisons of patients with diverse backgrounds, lacking a clear distinction between groups receiving several therapies across all studies. Despite efforts to ensure transparency and standardized reporting, a notable risk of bias persists. Caution is warranted in the interpretation and application of the data. This study was also subject to confounding variables and bias and is hampered by the quality of the pooled studies underlying the analysis. Across all studies, heterogeneity was frequently high, reflecting substantial variability in the reported incidence of chyle leak. This variability may stem from differences in patient populations, surgical techniques, perioperative management strategies, and definitions of chyle leak across studies.

Future research should focus on standardizing the definition and reporting of chyle leak to improve comparability across studies. High-quality prospective multicentre studies are needed to better characterize patient- and procedure-specific risk factors, and to develop targeted prevention strategies. The impact of surgical modifications, intraoperative lymphatic management techniques, and enhanced recovery protocols should be further explored to reduce the incidence and clinical burden of chyle leak following pancreatic surgery. Addressing these areas will help refine perioperative management and improve postoperative outcomes for patients undergoing pancreatic resections.

## Supplementary Material

zraf146_Supplementary_Data

## Data Availability

The data underlying this article are available upon reasonable request from the corresponding author.
