# Supplementary material for: Postoperative chyle leak after pancreatic surgery: scoping review
Source: BJS Open. 2026 Feb 9;10(1):zraf146. doi: 10.1093/bjsopen/zraf146 (PMC12884667; doi:10.1093/bjsopen/zraf146)
Supplement: zraf146_Supplementary_Data [file zraf146_supplementary_data.docx]

**Postoperative chyle leak after pancreatic surgery: scoping review**

Artur Rebelo^1^, Enzo Rauchbach ^2^, Jörg Kleeff^1,2^, Johannes Klose ^1^

^1^ Department of Visceral, Vascular and Endocrine Surgery, University Hospital Halle (Saale), Martin-Luther-University Halle-Wittenberg

^2^ Department of General, Visceral and Vascular Surgery, BG Hospital Bergmannstrost Halle (Saale)

Correspondence:

Artur Rebelo, MD, MHBA, FEBVS, FEBS, FACS

Department of Visceral, Vascular and Endocrine Surgery University Hospital Halle (Saale),

Martin-Luther University Halle-Wittenberg,

Ernst-Grube-Str. 40, 06120 Halle (Saale), Germany.

E-mail: [artur.rebelo@uk-halle.de](mailto:artur.rebelo@uk-halle.de)

**Supplementary Materials - Index**

| **Supplementary Methods** |  |
| --- | --- |
| Search strategy | *pag. 2* |
| **Supplementary Results** |  |
| Bias Analysis | *pag. 5* |
|  |  |
|  |  |

**Supplementary Methods**

A scoping review approach was selected as the most appropriate methodology for this study. Although a systematic review was initially considered, preliminary searches revealed considerable challenges in comprehensively identifying all relevant studies using standard systematic review methods. These challenges included inconsistent terminology across publications and databases, heterogeneity in study designs, populations, and reported outcomes, as well as the dispersion of relevant data across diverse sources, where the outcome of interest was often reported only as a secondary or incidental finding. As a result, a systematic review would likely have failed to capture the full scope of available evidence. In contrast, the scoping review methodology allowed for a broader and more flexible approach to evidence mapping, ensuring a more inclusive synthesis of the literature. This approach was therefore better suited to explore the extent and nature of existing research.

## Topic

1. Postoperative lymph/chyle fistula after pancreatic resection

## Main topic concepts definition

### P

| Pancreas  Pancreatic tumor |  |
| --- | --- |

### I

| pancreatic surgery |  |
| --- | --- |

### X

| Lymph fistula  chyle fistula |
| --- |

## Strategy

| 1 | P |  |
| --- | --- | --- |
| 2 | I |  |
| 3 | X1 |  |
| 4 | X2 |  |
| 5 | 1 AND 2 |  |
| 6 | 5 AND 3 |  |
| 7 | 5 AND 4 |  |

## Databases

- PubMed
- EMBASE
- Cochrane Library
- Web of Science Core Collection
- Clinical Trials.Gov
- ICTRP (WHO)

## Results report

The results were saved in Endnote and deduplicated. Some articles could still appear more than once.

The hits are sorted by database in Endnote. The PubMed hits were the first to be exported in Endnote. This makes them preferred for deduplication. In other words, in the case of duplicates, entries are removed from other databases.

The number of hits for each database in this report is based on its pre-deduplication status in EndNote.

## PubMed

### 1.P

| **"Pancreas"[Mesh] OR**  **"Pancreatic Neoplasms"[Mesh] OR**  Pancrea*[tiab] |
| --- |

### 2.I

| **"Surgical Procedures, Operative"[Mesh] OR**  **"surgery" [Subheading] OR**  **"Pancreatectomy"[Mesh] OR**  Pancreatectom*[tiab] OR  Operat*[tiab] OR  Surg*[tiab] OR  Excision*[tiab] OR  Dissection*[tiab] OR  resect*[tiab] OR  removal*[tiab] OR  ectomy[tiab] OR  ectomies[tiab] OR  Postoperat*[tiab] OR  Whipple[tiab] |
| --- |

### 3.X1

| **("Lymph"[Mesh] OR**  Lymph*[tiab] OR  Chyle*[tiab])  AND  **("Fistula"[Mesh] OR**  Fistula*[tiab] OR  Leakage[tiab]) |
| --- |

**Supplementary Figure 1: Search strategy.**

**Supplementary Results**

| Study | Selection (4) | Comparability (2) | Outcome (3) | Total Score (9) | **Evaluation** |
| --- | --- | --- | --- | --- | --- |
| Andrianello  et al 2020 | 4 | 2 | 3 | 9 | Good quality |
| Andrianello et al 2020 | 4 | 2 | 3 | 9 | Good quality |
| Assumpcao et al 2008 | 4 | 1 | 3 | 8 | Good quality |
| Aoki et al 2010 | 4 | 2 | 3 | 9 | Good quality |
| Augustinua et al 2023 * | 4 | 2 | 3 | 9 | Good quality |
| Bannone et al 2018 | 4 | 1 | 3 | 8 | Good quality |
| Bruna et al 20205 | 4 | 1 | 3 |  | Good quality |
| Cao et al 2020 | 4 | 2 | 3 | 9 | Good quality |
| Chen et al 2019 | 4 | 2 | 3 | 9 | Good quality |
| Dai et al 2020 | 4 | 2 | 3 | 9 | Good quality |
| Fang et al 2017 | 4 | 2 | 3 | 9 | Good quality |
| Gao net al 2019 | 4 | 2 | 3 | 9 | Good quality |
| Groen et al 2018 | 4 | 1 | 3 | 8 | Good quality |
| Gu et al 2020 | 4 | 1 | 3 | 8 | Good quality |
| Hackert et al 2017 | 4 | 1 | 3 | 8 | Good quality |
| Hackert et al 2018 | 4 | 2 | 3 | 9 | Good quality |
| Heckler et al 2020 | 4 | 2 | 3 | 9 | Good quality |
| Hartman et al 2024 | 4 | 1 | 2 | 7 | Good quality |
| Huang et al 2025 | 4 | 1 | 3 | 8 | Good quality |
| Hilal et al 2013 | 4 | 2 | 3 | 9 | Good quality |
| Ishii et al 2024 | 3 | 2 | 3 | 8 | Good quality |
| Lei et al 2024 | 3 | 2 | 3 | 8 | Good quality |
| Kim et al 2013 | 4 | 2 | 3 | 9 | Good quality |
| Klotz et al 2024 | 4 | 2 | 3 | 9 | Good quality |
| Kuboki et al 2013 | 4 | 2 | 3 | 9 | Good quality |
| Kulkarni et al 2020 | 4 | 2 | 3 | 9 | Good quality |
| Latenstein et al 2021 | 4 | 1 | 3 | 8 | Good quality |
| Lee et al 2021 | 4 | 1 | 3 | 8 | Good quality |
| Malik et al 2007 | 4 | 1 | 3 | 8 | Good quality |
| Mao et al 2020 | 4 | 2 | 3 | 9 | Good quality |
| Marchegiani et al 2018 | 4 | 2 | 3 | 9 | Good quality |
| Marchegiani et al 2018 | 4 | 2 | 3 | 9 | Good quality |
| Marchegiani et al 2018 | 4 | 2 | 3 | 9 | Good quality |
| Navez et al2019 | 4 | 2 | 3 | 9 | Good quality |
| N. de Graaf et al 2023 | 4 | 2 | 3 | 9 | Good quality |
| Nguyen et al 2024 | 4 | 2 | 3 | 9 | Good quality |
| Nguyen et al 2025 | 3 | 2 | 3 | 8 | Good quality |
| Nikel et al 2024 | 4 | 2 | 3 | 9 | Good quality |
| Noji et al 2012 | 4 | 1 | 3 | 8 | Good quality |
| Paiella et al 2018 | 4 | 2 | 3 | 9 | Good quality |
| Pamecha et al 2019 | 4 | 1 | 3 | 8 | Good quality |
| Pan et al 2015 | 4 | 2 | 3 | 9 | Good quality |
| Paniccia et al 2015 | 4 | 1 | 3 | 8 | Good quality |
| Parikh et al 2014 | 4 | 2 | 3 | 9 | Good quality |
| Partelli et al2017 | 4 | 2 | 3 | 9 | Good quality |
| Schmocker et al 2020 | 4 | 1 | 3 | 8 | Good quality |
| Schmocker et al 2020 | 4 | 2 | 3 | 9 | Good quality |
| Scholten et al 2019 | 4 | 2 | 3 | 9 | Good quality |
| Shyr et al 2020 | 4 | 2 | 3 | 9 | Good quality |
| Shyr et al 2021 | 4 | 2 | 3 | 9 | Good quality |
| Shyr et al 2023 | 4 | 2 | 3 | 9 | Good quality |
| Strobel et al 2017 | 4 | 2 | 3 | 9 | Good quality |
| Tjaden et al 2019 | 4 | 2 | 3 | 9 | Good quality |
| Van Beijsterveld et al 2020 | 4 | 2 | 3 | 9 | Good quality |
| Vinchurkar et al 2018 | 4 | 1 | 3 | 8 | Good quality |
| Yang et al 2015 | 4 | 2 | 3 | 9 | Good quality |
| Yang et al 2019 | 4 | 2 | 3 | 9 | Good quality |
| Yin et al 2018 | 4 | 2 | 3 | 9 | Good quality |
| Zhai et al 2019 | 4 | 1 | 3 | 8 | Good quality |

Table 1. Supplementary Table 1: Quality assessment for the risk of bias.


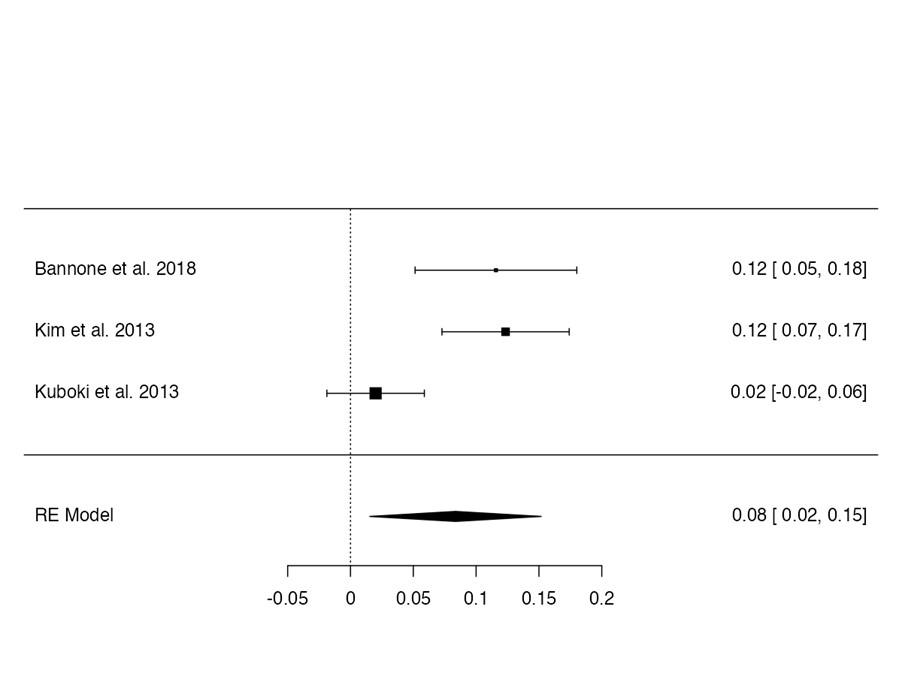


Figure 2: Pooled incidence of chyle leak after pylorus preserving pancreatoduodenectomy.


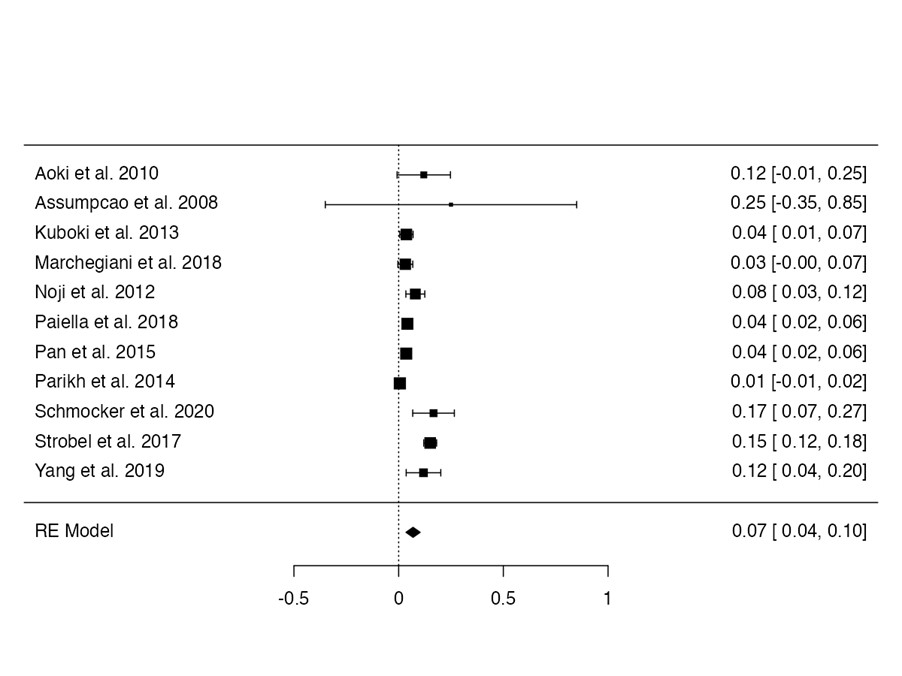


Figure 3: Pooled incidence of chyle leak after distal pancreatectomy.


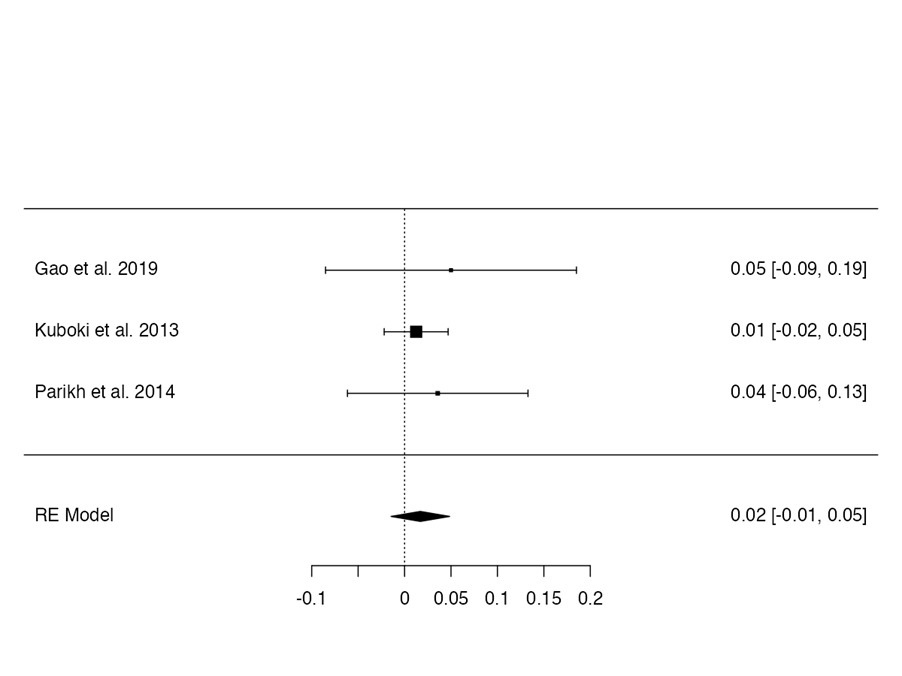


Figure 4: Pooled incidence of chyle leak after enucleation.


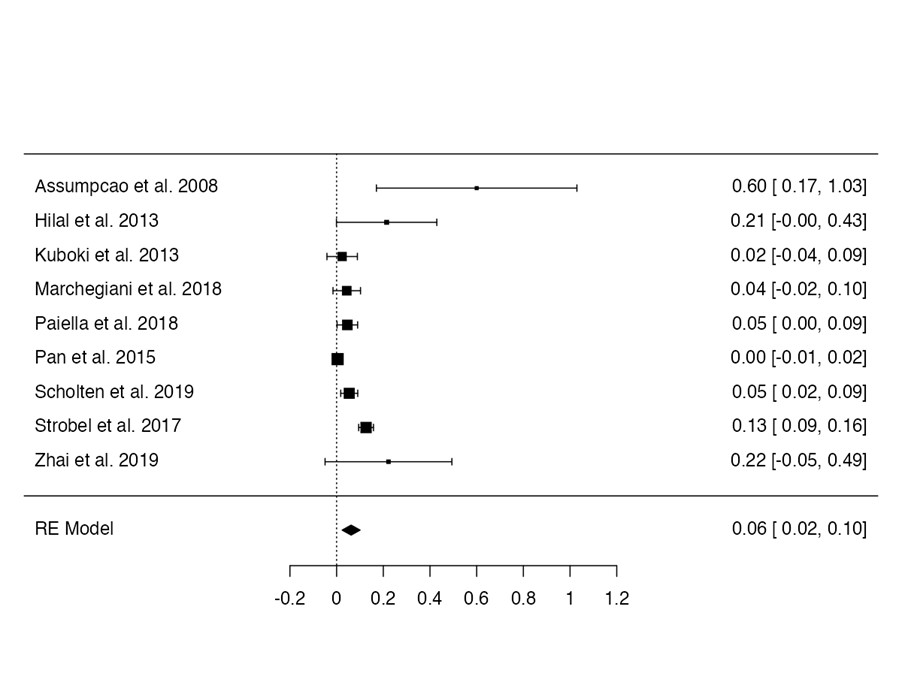


Figure 5: Pooled incidence of chyle leak after total pancreatectomy.
